# Evolving a Field: Can Evolutionary Theory Provide What the Study of Human Evolution Requires?

**DOI:** 10.1002/ajpa.70127

**Published:** 2026-01-27

**Authors:** Charles C. Roseman, Benjamin M. Auerbach

**Affiliations:** ^1^ Department of Evolution, Ecology, & Behavior, School of Integrative Biology University of Illinois Urbana Illinois USA; ^2^ Department of Ecology and Evolutionary Biology The University of Tennessee Knoxville Tennessee USA

**Keywords:** biological anthropology, evolution, extended evolutionary synthesis, heritability, human evolution, ongoing synthesis, plasticity

## Abstract

The extended evolutionary synthesis (EES) is a school of thought that maintains that genetic determination and natural selection are over‐emphasized in the study of evolution at the expense of non‐genetic inheritance and processes of evolution beyond selection. Its proponents call for the de‐emphasis of genetics and the adoption of a broader model of inheritance that includes cultural and epigenetic transgenerational effects and strong adaptive phenotypic plasticity. Presenting itself as a radical alternative to what it claims is a rigid and ossified theoretical orthodoxy, the EES has lately gained considerable traction among scholars of human evolution, and a distinct sub‐branch of the EES unique to the biological anthropological study of human evolution has emerged (the EES in human evolution). To date, however, no direct comparison between the EES in human evolution and other contemporary evolutionary approaches has been attempted to evaluate whether the EES in human evolution affords researchers an edge in articulating good questions and structuring research programs to answer them. After reviewing the landscape of evolutionary theory, we evaluate whether the EES in human evolution is capable of delivering the processually pluralistic vision of evolution it has long promised and whether it brings something that the decades‐long ongoing synthesis (OS) of evolutionary theory since the modern synthesis does not. We then conduct a head‐to‐head comparison to evaluate the relative explanatory efficacy of the EES and our preferred OS theoretical framework on several issues of human morphological evolution. We demonstrate that evolutionary perspectives as drawn from the OS have a much more clarifying effect on the investigation of human evolution than their EES‐based competitor. Far from being a radical extension of evolutionary thought, the EES in human evolution offers little more than another idiom in which to tell adaptationist stories and triumphalist narratives of the ascent of humanity. Theory from the OS opens up new horizons of possible investigation of human evolution in a uniquely processually pluralistic and rigorous framework.

We confess from the outset that this is a bit of an unusual *Yearbook* paper, so we wish to be up front about our motivations for writing it. Biological anthropologists have both contributed novel concepts to and borrowed new ideas from evolutionary biology. The study of human evolution—especially the branches that task themselves with studying fossils, archeology, primate behavior, and primate comparative anatomy—has long contributed to evolutionary theory (e.g., Rogers and Harpending 1983; Cheverud 1984, 1988; Blangero 1990). Ideas developed during the twentieth century that were borrowed from evolutionary theory, for instance, included sociobiology (e.g., Leonovicova 1992; cf. Wind 1982), which was meant to revolutionize behavioral primatology, as well as punctuated equilibrium (e.g., Stanley 1992), heterochrony (Gould 1977; cf. Shea 1989), and cladistics (e.g., Wood and Chamberlain 1986; Tobias 1988; cf. Harrison 1993), which were supposed to be Promethean contributions to the study of the human fossil record. All have faded in disciplinary memory without bringing the promised illumination on vexing issues in human evolution (see, e.g., critiques in Trinkaus 1990; Pennell and Harmon 2013; von Cramon‐Taubadel 2019; Schroeder and Ackermann 2023). The considerable excitement generated in the study of human evolution by the extended evolutionary synthesis (EES) over the last decade or so constitutes a particularly notable and ongoing example in the field's borrowing of trendy ideas at the edges of evolutionary thought.

The EES in broader evolutionary biology (hereafter “the EES”) advertises itself as a revolution in evolutionary thinking (Pigliucci 2007; Laland et al. 2015; Müller 2017; cf. Travis 2010; Levis and Pfennig 2019; Uller et al. 2019). It maintains that the evolutionary theory of the modern evolutionary synthesis (MS) has changed little since it took its final form in the 1950s. Contributors to the EES contend that the MS mandates conceptual fealty to selectionism and genetic determinism and dogmatically calls for the exclusion of development, plasticity, and the co‐construction of organisms and their environments when explaining the diversity of life. Proponents of the EES claim that, by including these latter processes, their extended theory delivers a combination of novel insights and historically repressed ideas about non‐selective dynamics and non‐genetic variational properties of organisms (Arthur 2002, 2004; Jablonka and Lamb 2005, 2020; Pigliucci and Müller 2010; Dickins and Rahman 2012).

Ever watchful for novel evolutionary approaches, a growing number of scholars of human evolution have looked to the EES for inspiration. This cadre of thinkers has been attracted to the EES by what they see as the increased dynamism of an evolutionary worldview that includes processes beyond natural selection and causes of evolutionarily relevant variation beyond the genetic (see, e.g., arguments in Fuentes 2015 and Kissel and Fuentes 2021). They note that humans are long‐lived and obligately cultural beings who often shape the environments around them, leveraging this to argue that the EES provides the best framework for understanding changes in human conditions through time (see, for instance, Fuentes 2015, 2016, 2021; Antón and Kuzawa 2017; Zeder 2018; Kissel and Fuentes 2021; Murray et al. 2021; O'Brien and Bentley 2021; Prentiss 2021; Lala and O'Brien 2023; Stock et al. 2023, among many others). We call this branch of the EES the “EES in human evolution.”

The EES in human evolution separates itself from the previous revolutionary evolutionisms imported into human evolution through the way in which it structures research and makes its presence known in the literature. Where previous revolutionary evolutionisms inspired several fluorescences of empirical work because they brought with them new methods ready to apply to data (e.g., Harrison 1993; Strait and Grine 1999), the EES in human evolution, as we demonstrate in this paper, has inspired little analysis of biological data and no formal theoretical investigation. It has generated no shortage of literature, however, as it has been the topic of many expository papers that lately review the previous reviews. Moreover, the EES in human evolution has been the focus of workshops, conference symposia, and special issues of journals. In our reading of nearly the entire corpus of work in the EES in human evolution, however, we find that it has inspired little engagement with data, method, or models.

We first engaged with the EES in human evolution because we independently had been encouraged by peers to consider using the theory in our own work. We felt compelled to engage with it in earnest, however, after several graduate students under our supervision and the supervision of colleagues received anonymous reviews on manuscripts and applications for funding that told them that their theory was out of date and that the EES was the remedy for the shortcomings of their scholarship. We took this as an invitation for us to make a thorough review of, and enter into a dialogue with, the EES in human evolution literature.

Our engagement with the EES in human evolution and interactions with its proponents through the review process and in direct correspondence leads us to believe that something strange is going on in the study of human evolution. After reading the EES in human evolution literature in some depth, we were faced with the question of how a body of theory that inspires limited methodological, empirical, and formal theoretical contributions compared with other evolutionary theories can support the production of a large number of reviews and position pieces. How is it that enough experts in the field are so convinced that the EES in human evolution offers a paradigm‐shaking set of ideas that editors and review panel chairs think it is acceptable to hinder the progress of early career researchers in the name of reforming the field? What is the promise of the EES in human evolution and when can we expect it to deliver on this promise?

A paper about whether any part of the EES is a new and revolutionary way of thinking about evolution would be redundant. This issue has been debated many times before (e.g., Futuyma 2011, 2023; Laland et al. 2014; dos Reis and Araújo 2020). Rather than recapitulate these debates, we take a different tack and ask a series of questions about theory and the study of human evolution:

1. *How did we end up in this strange situation?* The EES in human evolution is one of many evolutionisms that have drawn the attention of scholars of human evolution. To understand how it may have become prominent enough to be regularly featured in publications and reviewer feedback in spite of its lack of concrete contributions to the field, we briefly discuss its history and how its proponents have argued why it is necessary.
2. *Is the EES in human evolution better at structuring a research program than alternative bodies of evolutionary thought and method?* We are interested in whether a theory provides a conceptual framework for the construction of good models that are internally consistent and can be used to produce interpretable predictions and to link theory with data in meaningful ways. For this reason, the best way to evaluate the conceptual tools they give us to answer questions is to perform a head‐to‐head comparison of the EES in human evolution with an alternative perspective on evolution, one informed by quantitative genetics and other elements of the ongoing modern synthesis. Issues of adaptation, genetics, and plasticity are prominent differences between the EES in human evolution and other ways of thinking about evolution, and so we will be focusing on these topics in our comparison.
3. *What theory should we be using and where can we get it?* We end by justifying the use of what is regarded as standard evolutionary theory in biology and identify places in which scholars of human evolution might be able to make a positive difference. In addition, we point the interested reader to freely available resources to help them build their knowledge in evolutionary theory.

Our aim is to place the issue of theory in the study of human evolution on a footing firm enough to yield productive discussions about how we can best address questions in ways that lead to good accounts of the dynamics of evolutionary processes. Like Arnold (2014), we think that there is value in disagreement among thinkers to build a stronger understanding of evolution. Such disagreement is a mark of a healthy field undergoing continuing synthesis. Critically evaluating whether bodies of thought purporting to change our understanding of evolutionary processes actually introduce new and constructive theory and method to the conversation is a crucial part of such an effort, and this paper is our contribution to the ongoing discussion.

## 1 How Did We End Up in This Strange Situation?

The modern synthesis (MS), initiated by research in the 1910s and 1920s (e.g., Fisher 1918, 1930; Haldane 1924, 1932; Wright 1931, 1932) and broadly encompassing the period from 1930 to 1950 (Provine 2001, but see Huneman 2019), has been long recognized as the foundation of modern evolutionary biology (Huxley 1942; Mayr 1993; Provine 2001; Washburn 1951). While numerous ideas emerged from the collective work of biologists during this period, their contributions were unified using predictive models with well‐defined assumptions and methods to understand how populations of organisms—whether modeled with allele frequencies, morphologies, or behaviors—changed in response to evolutionary processes. As highlighted by Smokovotis (1997), Arnold (2014), and Stoltzfus (2017), though, the synthesis was neither a single event nor characterized by harmony among its contributors. Arguably, dissension among contributors to the synthesis accelerated concept development and helped overcome theoretical inertia, contributing to an ongoing synthesis (OS) that continues today (Arnold 2014).

What qualities define the OS? Researchers using the framework of the OS have created evolutionary models that use parameters that estimate elements essential to understanding how processes can change populations over time—examples include inheritance of traits, population size, fitness, selection, and gene flow. These models are used to predict potential outcomes and to challenge these predictions with empirical data. What is essential to the success of these models is that they are well defined, such that they make precise predictions that can be productively challenged with empirical data. Moreover, these models increasingly incorporate approaches and data from a breadth of research areas, from genomics to comparative biology to macroevolutionary phylogenetics, yielding a field that is, as Arnold (2014) wrote, “broad and diverse.” The OS moniker was developed in response to the increased popularity of the EES in several evolutionary disciplines and is used as a shorthand means of pointing out that much of what the EES avers has been neglected has in fact been extensively studied in the supposedly moribund time between the establishment of the MS and the present. It does not denote anything resembling a cohesive school of thought. Rather, it is a means of gesturing to all the developments in evolutionary theory that the EES falsely insists did not happen and to recognize that evolutionary theory is anything but monolithic.

Over the past two decades, researchers within evolutionary biology and related fields have engaged in debates about whether the theoretical ideas of the ongoing modern synthesis need emendation (Futuyma 2017; Lala et al. 2024; Laland et al. 2014; Laland et al. 2015; Pigliucci and Müller 2010; Pigliucci 2007; Wray's comments in Laland et al. 2014). Researchers have highlighted developments resulting from studies in developmental biology, functional genomics and molecular evolution, and ecology, emphasizing the importance of phenotypic plasticity (Pigliucci 2001), epigenetic and inclusive inheritance (Jablonka and Lamb 2005), and niche construction (Odling‐Smee et al. 2003). We note that these are not new concepts; all have been under study within the OS since the 1980s (Kirkpatrick and Lande 1989; Lewontin 1983, 1998; Via and Lande 1985), if not long before (e.g., Waddington 1953). The argument that dominates the EES literature suggests that methods based on the modern synthesis exclude these processes, in part because development was not included in the MS as originally conceived (although development has been an important contributor since the 1960s as part of the OS), and in part because some discoveries were not available until technological developments allowed for their observation (Lala et al. 2024).

The EES, introduced in the mid‐2000s (Pigliucci 2007), is not a single idea, but it is unified by a claim that evolutionary theory based on the MS lacks the insights that would be achieved by incorporating processes derived from development, biochemistry, and biogeography. A common concern argued by writers in the EES is that processes beyond those identified in the MS are important parts of evolution (e.g., Baedke et al. 2020; Jablonka and Lamb 2020), with some asserting a need for urgent reform of the MS (Laland et al. 2014). Evolutionary biologists outside the EES responding to these position papers make a case that more empirical evidence and theoretical work are required to support the argument for the incorporation of the inchoate ideas presented in the EES into the larger evolutionary framework (Futuyma 2017; Wray's comments in Laland et al. 2014). In no instance do the latter group of researchers argue that there is no room for the processes cited by Pigliucci and Müller (2010) or Laland et al. (2014). As stated by Wray (Laland et al. 2014):

All four phenomena that Laland and colleagues promote are ‘add‐ons’ to the basic processes that produce evolutionary change: natural selection, drift, mutation, recombination and gene flow. None of these additions is essential for evolution, but they can alter the process under certain circumstances. *For this reason they are eminently worthy of study…*. We, too, want an extended evolutionary synthesis, but for us, these words are lowercase because this is how our field has always advanced. (164, emphasis ours)

Like any set of ideas put forward as hypotheses, we agree with Wray that the evolutionary role of processes championed within the EES should be assessed, but they should not be assumed to replace the many processes that have been studied over the last century as part of the MS and OS. To that point, the last sentence in this quote echoes the idea that evolutionary theory is a product of an ongoing synthesis and not a rigid system that has suppressed change since the 1950s.

For example, issues important to EES practitioners such as developmental bias (Arthur 2004; Hordijk and Altenberg 2019; Levis and Pfennig 2019) and the ways in which environment‐organism interactions can enable or constrain evolutionary change (see, e.g., Love and Wagner 2022) have received considerable attention in the larger OS literature. In no case, however, has the empirical or theoretical study of these EES‐emphasized dynamics supplanted evolutionary theory generated during the ongoing synthesis. Rather, studies like Love and Wagner (2022) have augmented an understanding of how evolutionary processes (e.g., selection) may act on regulatory variation introduced through environmental interactions in a manner that seamlessly alloys them with the basic evolutionary principles of the OS.

Scholars of human evolution in its traditional anthropological home have recently engaged in the EES, both in individual papers starting in the early 2010s and more recently in two sets of special issues: *Evolutionary Anthropology* (2021, Issue 1) and *Paleoanthropology* (2023, Issue 2). A theme of these papers is an effort to find evolutionary processes that explain purportedly exceptional traits (e.g., brain size, degree of morphological variation, or complex social behavior), coupled with the argument that evolutionary processes modeled by theory in the OS are insufficient to provide these explanations. Collectively, these EES in human evolution publications do not discuss the breadth and depth of the work in the OS, but instead suggest that the MS is ossified, if not fossilized, and that unnamed supporters of the MS are wedded to a dogma that is allele‐ or gene‐focused and allows no room for constructive processes that occur during development or over the lifespan of organisms to influence evolution. It is clear from the quote we provide above from Wray (Laland et al. 2014), though, that even among those cited as the most dogmatic in support of not changing the MS (authors like Gregory, Wray, and Futuyma; see Zeder 2018, 268), the majority of evolutionary biologists are more interested in a dialogue and empirical tests to ascertain the roles EES‐emphasized processes may play in organismal evolution than would be expected from zealous defenders of a dogma.

Echoing our colleagues in evolutionary biology, we are concerned that excitement among anthropologists for ideas in the EES is tied to a false narrative in which modern evolutionary biologists deny the importance of non‐genetic variation or of processes beyond natural selection acting on genetic variation as being drivers of evolution. Proponents of the EES concomitantly ignore the fact that work within the OS has not stopped since the early 1960s (Arnold 2014). For example, proponents of the EES in human evolution proclaim that their preferred theoretical framework allows for a processual pluralism that includes processes beyond natural selection to be incorporated into the study of human evolution. We wholeheartedly endorse processual pluralism (see Auerbach et al. 2023). Yet instead of, for example, invoking evidence for the importance of gene regulation in the facilitation of and establishment of constraints on phenotypic evolution (e.g., Young et al. 2022, Senevirathne et al. 2025) or the important causal roles played by neutral processes in phenotypic evolution (well explored, among many others, by Ackermann and Cheverud 2000; Roseman and Auerbach 2015; Roseman and Weaver 2007; Schroeder and Ackermann 2023; Schroeder and von Cramon‐Taubadel 2017; von Cramon‐Taubadel 2009), proponents of the EES in human evolution claim, without evidence, that plasticity and non‐genetic explanations are the more important processes by which organisms evolve.

This raises one of our core questions: Does the EES in human evolution provide a constructive alternative to understanding evolution, especially as applied to humans? Answering this question is best accomplished through a direct comparison of the efficacy of the EES in human evolution and the OS when it comes to structuring and answering questions about human evolution. Does such a comparison support the supplantation of OS‐based approaches in the study of human evolution by those derived from the EES?

## 2 Is the EES in Human Evolution Better at Structuring a Research Program Than Alternative Bodies of Evolutionary Thought and Method?

As we note above, proponents of the EES in human evolution distinguish it from the remainder of evolutionary thought principally by highlighting the importance of evolutionary processes beyond natural and sexual selection; non‐genetic influences on variation; and the roles of plasticity and organism‐environment interactions. By the EES in human evolution proponents' reckoning, more traditional bodies of evolutionary thought are neglectful of these considerations. Rather than asking whether the EES in human evolution is novel or whether the remainder of evolutionary thinkers are neglecting and suppressing important ideas, we examine whether a researcher can structure a productive research program better with the EES in human evolution than with other alternatives.

To accomplish this, we first evaluate whether the EES in human evolution is as processually pluralistic in its theory as it advertises, followed by a similar theoretical interrogation of its claims about plasticity and genetics. We will be upfront about our conclusion, to which we arrive after comparing the efficacy of the EES and OS to address human evolutionary questions in the coming pages: the EES in human evolution does not provide an improved framework for asking questions about evolution in humans or any other group of organisms. For one, as we will demonstrate, the ideas of the EES in human evolution do not provide well‐defined models that yield precise expectations. Moreover, its treatments of evolutionary process, plasticity, and genetics are unclear and often share the same basic argumentative framing as those found in evolutionary psychology and race science.

Our interrogation of the EES in human evolution and other bodies of evolutionary thought is largely theoretical. This is because there are precious few examples of EES theory being applied to empirical problems in the study of human evolution. Our exercise here is to discuss how proponents of the EES in human evolution engage with evolutionary models and ideas that have been established in the OS, so we can better understand the positions held within the EES as applied to human evolution. We follow up on this review with a head‐to‐head comparison of the efficacy of the two bodies of theory by re‐analyzing two of the few studies on plasticity and adaptation in human evolution that apply EES in human evolution thinking to data. In each case, evolutionary theory informed by OS models is much more capable of identifying appropriate questions and structuring their investigations than that provided by the EES.

### 2.1 Is the EES in Human Evolution Processually Pluralistic?

In a call for a new approach, EES in human evolution papers (e.g., Fuentes 2015; Antón and Kuzawa 2017; Fuentes 2021) emphasize a processual pluralism that involves the inclusion of several evolutionary processes beyond the five most often cited in standard evolutionary theory literature. What counts as an evolutionary process in the EES in human evolution is not expressed with much precision. Niche construction and the co‐construction of organisms and environments are gestured at by the EES in human evolution, but there has not been much of an effort to operationalize these concepts such that they may be directly applied to problems in human evolution. Since proponents of the EES in human evolution and practitioners of OS approaches to evolution all hold that the evolution of genes and genomes is important, there is a simple test of processual pluralism that we apply to evaluate EES in human evolution claims: Does the EES in human evolution describe a diversity of evolutionary processes in ways that other schools of thought—especially the OS—do not?

#### 2.1.1 Evolutionary Processes Not Considered in the EES

In the most restrictive definition of biological evolution—change in allele frequencies over time, where an “allele” is a variant of a nucleotide sequence—five processes are typically identified as being able to affect evolutionary change: mutation, random genetic drift, gene flow, recombination, and selection. If you consult any one standard evolutionary theory source, you are likely to find only four of these mentioned, as different schools of thought tend to leave out either gene flow or recombination (e.g., Lynch 2007). Note that whether to include gene flow, recombination, or both processes in the basic theory of biological evolution is an example of theoretical heterogeneity in a field that is described as an intellectual monoculture by proponents of the EES in human evolution. We will consider the first four of these processes before focusing on selection and adaptation.

##### 2.1.1.1 Mutation and Recombination

Mutation and recombination are the two evolutionary processes that can generate novel genetic variation in a population. Mutation is any of a large variety of changes to a sequence of nucleic acids that occur because of lack of complete fidelity in the reproduction of DNA during cell replication. Recombination refers to a change in the collection of alleles linked to one another in a part of a genome (i.e., a haplotype). While none of the alleles at individual parts in the genome are novel, haplotypes arising from recombination may be novel combinations of alleles across loci. Recombination can break down linkage disequilibrium—the tendency for alleles to be non‐randomly associated because they are inherited together (i.e., they are linked)—leading to changes in haplotypic frequencies in a population and thus results in a kind of evolution. Throughout the models used in the OS, mutation and/or recombination are either explicit parameters or are modeled to be in equilibrium with other evolutionary processes (e.g., mutation‐drift balance).

Mutation and recombination make vanishingly few appearances in the EES in human evolution. The one paper in the EES in human evolution literature that explicitly engages with genetics and genomics makes no mention of recombination, and mutation makes but a fleeting appearance in a list of evolutionary processes (Fuentes 2021). Neither process occupies much of the attention of the remainder of the anthropological EES literature and is only mentioned in perfunctory descriptions of the evolutionary process before being set aside and ignored.

##### 2.1.1.2 Random Genetic Drift

Random genetic drift, the stochastic effect of sampling on allele frequencies in a finite population, is a foundational theoretical principle for molecular evolution, evolutionary genomics, population genetics, and the study of phenotypic evolution alike. Ranging from the basic structure of the eukaryotic genome (Lynch 2007) to the evolution of the form of the human head (Ackermann and Cheverud 2004; Roseman 2016; Schroeder et al. 2014; Weaver and Stringer 2015) and postcrania (Cho et al. 2022; Roseman and Auerbach 2015; Savell et al. 2016; Savell et al. 2022), random genetic drift plays a key role in various evolutionary transitions that have long been the preserve of adaptationist speculation.

Despite the revolutionary role of neutral theory in evolutionary biology, random genetic drift (and neutral mutation) receives almost no mention in the EES in human evolution beyond descriptions of evolution as a whole (exceptions include Kissel and Fuentes 2021; Prentiss 2021; Van Arsdale 2017). While genetic drift is considered a potential explanation for some of the evolution of morphology in the genus *Homo* (Van Arsdale 2017), an evolutionary model is not incorporated into the analysis of the data, and the conclusion instead relies on random genetic drift as a post hoc explanation for a pattern. Such pattern‐based argumentation is useful for establishing hypotheses to be tested using evolutionary models but should not be mistaken for the actual analysis of potential evolution; for example, studies of variation in body form among recent humans (Auerbach 2010, 2012; Trinkaus et al. 2014) established patterns later tested using evolutionary quantitative models (Roseman and Auerbach 2015; Cho et al. 2022; Savell et al. 2022). Similarly, Prentiss (2021) consider the literature on the apparent neutrality of morphological change in humans through the Pleistocene as a part of a larger review, but do not engage in any EES in human evolution‐informed analysis of data. Other contributions to the EES in human evolution literature review the effects of random genetic drift‐like stochastic processes on cultural evolution (Kissel and Fuentes 2021), but none present empirical or theoretical work linking the evolution of the human organism and culture, material or otherwise, that feature stochastic dynamics.

##### 2.1.1.3 Gene Flow

Beyond a generic anthropological commitment to highlighting the ways in which humans throughout the world have been interconnected by migration, the effects of gene flow and hybridization are not included in the EES in human evolution literature. In our review of EES in human evolution papers, we found no substantive engagement with gene flow as a source of variation or evolutionary change. Hybridization is a conspicuous omission from the EES in human evolution as its effects highlight interactive processes that have been too often neglected by large parts of human genetics and identify a principal shortcoming of a gene‐focused view of evolution (Ackermann et al. 2016; Buck et al. 2025).

#### 2.1.2 Plasticity as a Concept in Biology and Evolution

We have mentioned plasticity a few times in passing, but the concept deserves close scrutiny before we consider how work in the OS and EES addresses natural selection. The most general case of plasticity is “developmental plasticity,” which has a variety of definitions, all of which denote the tendency for an organism to develop different aspects of phenotype in response to different prevailing environmental conditions, whether they be internal or external to the organism (West‐Eberhard 2005). “Developmental plasticity” is deployed in several ways depending on the context. These include the ways in which early life experiences influence later developmental outcomes (Lea et al. 2017) and the ways in which experience can change neural connectivity as a part of learning (Calvey 2017; Jablonka 2017; Miranda 2019). Not all applications of the term refer to genotype. When differential outcomes in response to the environment are reckoned with respect to a single genotype, plasticity is generally referred to as “phenotypic plasticity.” The tendency of an organism with a given genotype to display phenotypic plasticity across a range of environments is described by its “norm of reaction” (Woltereck 1913).

Anchoring plasticity with respect to a genotype allows plasticity to be distinguished from developmental noise, which is variation arising from the randomness inherent to organic processes (Gavrilets and Hastings 1994). This includes small variations in local rates of cell division during development and random differences in the number of organelles among daughter cells after cell division, which can have effects on larger scales later in life. Developmental noise can vary in magnitude across traits within groups of organisms, across groups of organisms, and appears to evolve in its own right (Gavrilets and Hastings 1994; Richard and Yvert 2014; Yampolsky and Scheiner 1994). We note that developmental noise is not regarded as phenotypic plasticity because it does not have an effect on the average phenotype associated with a genotype when properly scaled. In making this distinction between plasticity and noise, “phenotypic plasticity” relates plasticity and genetics in a manner that “developmental plasticity” does not.

None of this treatment of plasticity is controversial as there is widespread recognition that it is a common feature of organisms. Arguments tend to be over the degree to which it is manifest in any given situation rather than whether it occurs at all. That plasticity can evolve is not particularly controversial either, as there is a considerable amount of theory about how it might do so, and it is a topic of considerable importance in plant and animal breeding (see Lynch and Walsh 1998, Ch. 22, for a digestible summary). Disagreements abound, however, when we move to issues of whether plasticity is adaptive.

“Adaptive plasticity” is the tendency for an organism to display plasticity that reacts to different environments in a manner that increases its fitness—a concept we explain in the next subsection—over and above what is expected for that phenotypic state in an alternate environment. Systems in which adaptive plasticity has been well studied include the response of *Daphnia* to the presence of predators (Nagano et al. 2023) and in domesticated cereals (Brooker et al. 2022). The evolution of adaptive plasticity depends on the availability of genetic variation in the form of variation in the norms of reaction themselves. To establish that plasticity is adaptive, we need to demonstrate that variation at some level of organization is attributable to plasticity, that plasticity tends to result in phenotypic outcomes that are fitness‐enhancing in different environments, and that the plasticity evolved by natural selection acting on the appropriate genetic variation in plasticity. Explanations that appeal to adaptive plasticity as the reason for patterns of change over time within a species or clade without directly addressing these issues are products of idle speculation.

#### 2.1.3 Equating Evolution With Adaptation

How does the EES in human evolution address natural selection? In many ways, selection and adaptationism appear to be the principal motivators for the critiques by proponents of the EES in human evolution. They often present phenotypic plasticity as an alternative process by which variation or adaptive evolutionary change occurs. However, adaptation is still the focus of the argument. Thus, while multiple processes are proposed by the EES in human evolution, they address only adaptation and not evolution in general. This makes the EES in human evolution a kind of expanded adaptationism and not a theory of evolution.

Selection is a kind of variation in fitness, and the nexus unifying fitness and function is conventionally regarded as a key part of adaptation (Arnold 1983). Fitness itself is a measure of how an individual with a given variant in a population (e.g., a particular genotype or phenotype) is expected to survive and reproduce. If the variant with the highest fitness is closest to the mean of that genotype or phenotype for a population, the result is stabilizing or purifying selection. If a variant with the highest fitness is different from the mean, then over successive generations the population mean will shift toward the value of that variant (i.e., directional selection). This assumes that variation in a genotype or phenotype has a causally based correlation with fitness, an assumption that needs to be tested within an evolutionary framework that allows us to account for differences in fitness.

The EES in human evolution almost always describes alternative evolutionary processes as substitutions for or elaborations on processes of adaptation by selection. There are examples of this in most EES in human evolution papers we reviewed. For instance, in these papers, evolution is equated with fitness or function, as “[i]t is absolutely clear that there are multiple modes by which evolutionarily relevant – that is, fitness influencing or function influencing changes across time in populations – processes occur” (Fuentes 2021, 331). Taking this at face value would mean that we would have to regard, for example, random genetic drift as a process that is not evolutionarily relevant because it does not relate directly to fitness. Likewise, Antón and Kuzawa (2017, 3), in exploring phenotypic traits they argue to be the products of phenotypic plasticity, noted that “because natural selection can only operate on existing phenotypes, these plasticity‐induced trait configurations are the raw variation that is subjected to selection, and that eventually facilitates genetic adaptation.” Thus, this argument suggests that plasticity generates the only evolutionarily consequential variation (rather than mutation, gene flow, or recombination) and that only variation that leads to adaptation is important to understand evolution.

The EES in human evolution emphasizes adaptation and selection to the near total exclusion of other processes, even if adaptation has been decoupled from natural selection acting on heritable *genetic* variation. In multiple publications, processual pluralism is gestured at through phenotypic plasticity. But how plasticity, a developmental dynamic that plays out during the lifetime of an organism, relates to evolution, a process that takes place over lengths of time that can exceed the lifetime of an individual, is not clear in this formulation.

A few examples drawn from the EES in human evolution literature demonstrate this commitment to adaptation. Antón and Kuzawa (2017, 3) also write that “[e]nvironmentally induced phenotypes may ‘lead the way’ to more gradual genetic adaptation through several processes, which have been labelled ‘phenotype‐first’ evolution.” This sentiment is echoed in Zeder's (2018) discussion of how phenotypic plasticity can permit changes in the variational properties of traits in response to changes in the environment, which then aids in the adaptation of a group to novel environmental conditions:

[C]ryptic variation, accumulating under conditions of relaxed selection during periods of environmental stasis, is especially likely to be activated when organisms are exposed to novel environments, resulting in rapid adaptation to new conditions. (Zeder 2018, 271)

Both of these papers refer to ideas associated with genetic assimilation (Waddington 1953, 1956, 1961) or genetic accommodation (West‐Eberhard 2005). However, neither considers the large body of literature in the OS that investigates how phenotypic plasticity can evolve by and affect the operation of natural selection (e.g., Lande 2009, 2014; Scheiner et al. 2017).

A close reading of the EES in human evolution confirms that its version of “evolution” is an extended adaptationism as opposed to being a pluralistic extension of evolutionary theory. For example, Stock et al. (2023) present their EES‐motivated intervention into issues of recent human skeletal variation by framing it as fundamentally a problem in adaptation:

…evidence for phenotypic and behavioral diversity within the genus *Homo* to evaluate the hypothesis that our evolution was characterized by a shifting distribution of adaptation across different systems described by the EES. We define and apply a model that we term ‘distributed’ adaptation, where mechanisms of both plasticity and culture serve to accommodate environmental variability in ways that are more rapid than genetic adaptation, thus distributing selection across a range of adaptive systems. (206)

This approach within the EES stands in stark contrast to recent developments in the OS tradition in human evolution that offer genuine alternatives to a‐processual adaptationism (Auerbach et al. 2023; Schroeder and Ackermann 2023; von Cramon‐Taubadel 2019).

It is important to remember that all evolution by natural selection is phenotype‐first in nature because genetic effects on fitness are always phenotypically mediated. This includes features of genomic composition and structure such as codon bias (Hershberg and Petrov 2008). Mutation, recombination, and gene flow, on the other hand, are inherently genotype‐first because they are identified with respect to their effects on allele frequencies alone. For instance, gene flow cannot take place between two groups if the allele frequencies are identical even if the groups are exchanging migrants. Individuals may move and reproduce in a different group, but there would be no gene flow as allele frequencies do not change.

Adaptive plasticity certainly occurs (e.g., Becker et al. 2022). But the best theories of the evolution of adaptive plasticity model it as the product of selection over generations on varied phenotypic outcomes that need to be the product of certain kinds of genotype‐by‐environment interactions (e.g., Lande 2009; Sommer 2020). A general capacity to exhibit plastic responses to changing environments, such that organisms tend to adapt to different circumstances would require a great deal of selection on the right kind of variation to evolve. Thus, casual appeals to adaptive plasticity have the effect of doubling down on adaptationist and selectionist explanations as opposed to liberating investigations from their grip.

Any way that development, environment, and plasticity figure into these accounts, our review of the EES in human evolution literature reveals that selection and adaptation are what are promoted as genuine evolution in that school of thought. Discussions of the other four evolutionary processes, as we explored above, are rare exceptions and often not substantive parts of either critiques or data analysis. While the notion of heredity is expanded to include variation arising from non‐genetic influences, such as culture or enduring modifications to environmental conditions, understanding adaptation by selection is the narrow evolutionary focus of the EES in human evolution.

#### 2.1.4 Plasticity, Genetics, and Human Evolution

In our review of EES in human evolution publications, we were also struck by its adherence to the false opposition between heritability and plasticity. Heritability is a statement about variation in a population that expresses the proportion of the phenotypic variance that is made up by additive genetic variance. Additive genetic variance is the variation that is manifested as resemblances among relatives that varies as a linear function of the relatedness among organisms, independent of how environments are distributed. The primary use of additive genetic variance is in predicting the degree to which a population of organisms will exhibit an evolutionary response to selection, random genetic drift, or gene flow given that environmental conditions remain roughly constant across generations. Phenotypic plasticity expresses the tendency of an individual bearing a particular genotype to exhibit different phenotypic outcomes in different environmental conditions.

More succinctly, heritability is concerned with what is actually manifest in a population at a given time and a given environmental context in terms of how strongly phenotypic variation is structured by relatedness. Plasticity is concerned with how the phenotypic states of individuals with particular genotypes vary across different environments. As such, heritability and plasticity describe distinct properties of how organisms vary, and the links between the two concepts and their statistical expressions are not straightforward. Disentangling the effects of plasticity from genetic effects is difficult in the extreme, even when heritability is known with precision. Outside of well‐controlled experimental settings, uniquely identifying genetic and environmental effects is even more difficult and requires the application of combinations of polygenic risk scores as instrumental variables, randomized controlled trial designs, pedigrees containing many classes of relatives, and sophisticated statistical methods.

While these challenges are well appreciated in the broader evolutionary and complex trait genetics communities, which have developed theoretical and methodological answers to many of the challenges (e.g., Roff 1997; Scheiner 2002; Scheiner and Lyman 1989), the EES in human evolution literature makes limited mention of them. The majority of the studies cited to support claims of plasticity, trans‐generational environmental effects, and epigenetic influences in the EES in human evolution implicitly assume that parents and their offspring are unrelated to one another to reach the conclusion that resemblances between mothers and offspring are attributable to plastic responses to environmental influences as opposed to genetic effects (e.g., Chung and Kuzawa 2014; Jasienska et al. 2006; Kuzawa et al. 2010). These studies have the same problem as human behavioral genetics studies that depend on data from twins (Anholt and Mackay 2009), in that they do not control for the fact that genetic and environmental causes tend to be confounded in most human reproductive and familial situations. As such, they are useless for discriminating between environmental maternal, common familial environmental, and genetic effects.

Beyond the issue of disregarding the empirical and methodological problem of confounding among genetic and environmental effects, the assertion that to be genetic is to be determined and to be plastic is not to be genetic is repeated throughout the EES in human evolution literature. For example, Wells and Stock (2011) reviewed the heritability of traits known to have undergone strong secular changes over the last century or two (e.g., stature, age at menarche, and birth weight, among others) and demonstrated that there was essentially no relationship between the magnitude of the secular trend in and the heritability of a characteristic. They use this to claim, “there is evidence for substantial plasticity in these traits, indicating that a significant component of variability is not controlled directly by genomic variation, and therefore heritability could have been overestimated” (422). Likewise, Stock et al. (2023) state

…the role of phenotypic plasticity in human evolution and its contribution to fossil hominin morphology is poorly understood. This is due, in part, to a paradox in our knowledge of the mechanisms influencing the human phenotype. Many aspects of human phenotypic variation are assumed as being highly heritable based on twin or sibling studies, although many of the most ‘heritable’ traits are also highly plastic during growth and development. (208–209)

The scare quotes serve as extra emphasis on the acceptance of the false dichotomy pitting heritability against plasticity. These sentiments are echoed by Antón and Kuzawa (2017, 2) who write, “a key prerequisite [of fossil Hominin taxonomy] was determining the relationship of new [fossil] discoveries to previously known species by using aspects of fossil anatomy that were inferred to be heritable (i.e., genetically determined).”

Genetic traditions that emerged as a part of the OS are unanimous with respect to the relationship between the heritability of a trait and its capacity for plasticity: There is no relationship. (We explained why above.) As such, there is no paradox. High heritability gives no indicator that a trait cannot display plastic responses across a range of environments, and traits with low heritability might very well be stubbornly resistant to environmental intervention.

By conflating heritability with genetic determination and a lack of plasticity, the EES in human evolution's position is indistinguishable from that of the genetic determinists and race scientists in the psychology literature who insist, or at least imply, that pseudo‐characteristics like psychometric intelligence are highly heritable and thus not capable of exhibiting plasticity. As is the case for the hereditarian race science view of genetics, the EES in human evolution position is not consistent with the over a century of work in the literature that forms the intellectual roots of the OS and research that continues as a part of the OS.

### 2.2 Test Cases for the Relative Efficacy of the EES and OS

From the review of the literature in the EES in human evolution, we find: (1) an argument that variation has arisen through processes other than mutation, gene flow, or recombination (i.e., through adaptive plasticity), (2) neutral evolutionary processes are often unincorporated into evolutionary arguments, and (3) an overemphasis on adaptation. But this does not help us understand the quality of the scientific questions and analyses the “add‐ons” the EES in human evolution provides in evolutionary research. Ordinarily, we might accomplish an evaluation of this by reviewing a series of papers with shared applications of theoretical frameworks to empirical problems. In the case of the application of the EES to problems in human evolution, however, there are few examples of connections between theory, method, and data, as the EES in human evolution literature consists mostly of several generations of review papers. Thus, we use two of the few EES in human evolution publications that provide both data and models for in‐depth examinations and partial re‐analysis. Both studies focus on plasticity and adaptation in human evolution, one focusing on worldwide variation in human body form (Stock et al. 2023) and the other on the evolution of large brains during human evolution (Antón and Kuzawa 2017). These studies are an impetus to critically evaluate how evolutionary processes are examined across the EES in human evolution literature.

#### 2.2.1 Plasticity and Adaptation of Human Body Form

As a first comparison of how well the OS and EES equip researchers to answer questions about human evolution, we focus on the analysis of global variation in human body form and limb proportions^1^ presented in Stock et al. (2023). In it, the authors draw on ideas from the larger EES to argue that much of the variation in body form among recent humans and fossil hominins is a product of plasticity during growth and development. In their account, phenotypic plasticity is a key source of variation as evidenced through (1) the “rapid emergence of regional phenotypic variation [stature and body mass] within a species” and (2) shifting patterns of limb proportions over primary growth among individuals living at latitudes over 50 degrees north. In each case, the differences among groups are claimed to arise from adaptation to life in different local conditions via phenotypic plasticity. The “adaptation” part is key. It is not simply that there is a plastic change in limb proportions or other aspects of body form. Rather, it is that there is an evolved propensity for plasticity to produce responses that tend to allow the organism to flourish.

We identify three topics raised in the Stock et al. (2023) analysis where head‐to‐head comparisons between EES in human evolution and OS approaches provide an opportunity to evaluate the relative ability of each approach to identify productive questions and deploy theory and method to answer them. Using published results, we evaluate the claim that humans are particularly variable (meaning able to vary in response to developmental or evolutionary processes) in body form as compared to other organisms. This claim serves as a warrant for the argument that an EES‐based approach is necessary for understanding the evolution of human body form variation. We further examine how the propensity for humans to respond to environments in ways that are argued to be adaptive has been theorized. In our review of the EES in human evolution literature, strong insistences are made about how phenotypic plasticity allows humans to adapt rapidly to novel conditions. This assertion, which we coin as a *meta‐adaptive capacity*—an adaptation that affords more adaptations, even in response to factors to which a lineage has not been exposed—is a radical proposal, departing even from the most relaxed interpretations about evolvability as discussed, for example, in Hansen et al. (2023). Finally, we examine the tendency for work in the EES in human evolution not to consider that evolved genetic differences among groups can result from historically contingent evolutionary dynamics that exhibit path dependence.

##### 2.2.1.1 Do Humans Tend to Vary More Than Other Animals With Respect to Body Form and Limb Traits?

Motivating many arguments made in the EES in human evolution is a contention that humans tend to show high variation in body form. For example, Stock et al. (2023, 213) claim that “[m]odern humans are highly variable in body size, variation that is achieved through a combination of genetic variation and developmental plasticity in response to environmental stimuli.” The assertion is that the observed variation in humans is much greater than that found in other organisms or, as compared to some theoretical ideal, is treated as axiomatic in the EES in human evolution, though both the empirical and theoretical premises of the argument are unverified.

Relating evolutionary processes to developmental dynamics is key for understanding the disposition of variation, which is indispensable for modeling how evolution takes place over the time scales on which human evolution took place (Agosto and Auerbach 2021; Atchley and Hall 1991; Auerbach et al. 2023; Cheverud 1984; Senevirathne et al. 2025; Young et al. 2019, 2022; Wall‐Scheffler et al. 2020, 99‐107). Unguided by theory, however, no empirical exercise can provide anything resembling a dispositive, or even a marginally informative, result. This applies to the Stock et al. (2023) analysis as while they attribute variation to “a combination of genetic variation and developmental plasticity in response to environmental stimuli” (p. 9), they do not relate their claims about variation in a theoretical framework that makes explicit predictions about how variation arises in a group or accumulates between groups and allows us to compare models and test hypotheses. Rather than ignoring these kinds of problems as suggested by proponents of the EES in human evolution, the OS literature is replete with theory that allows for this kind of model building and comparison.

The simplest, although least informative, question to address is the brute empirical issue of whether humans tend to display unusually high levels of variation. Often, researchers do this using a mean‐standardization approach in which variation is calculated within local groups and expressed as a coefficient of variation (*CV*), which standardizes the variation by the mean to make expressions of variation comparable over a wide range of values (see Lobry et al. 2023 for a critique of its use). This is similar to an evolvability framework in which variation (additive genetic or phenotypic variance, typically) is standardized by the square of the mean (Hansen 2023; Houle et al. 2011). The former quantity is familiar across both social and natural science disciplines, although the latter has more interpretable statistical and evolutionary properties (Pélabon et al. 2020).

With respect to the direct comparison of phenotypic variation in the absence of a theory of variation, humans do not tend to vary more than other animals in body length (stature in humans) or body mass (McKellar and Hendry 2009). Higher variation in humans is also not the rule when it comes to postcranial features, depending on the scope of the comparison. For example, in individual linear metrics describing pelvic form, for instance, humans have lower variation than is found in other primate species (Grabowski et al. 2011; Young et al. 2022).

Regarding variation within‐groups, models based on neutral theory seem to fit some aspects of phenotypic variation in humans. Morphological variation in the cranium is correlated with the amount of presumably neutral genetic variation in local groups from across the world (Roseman 2004, 2016; Katz et al. 2016). There is a long tradition of struggling with the complex and difficult problem of understanding ways in which differences in development may shape the disposition of variation, whether from genetic, environmental, and interactive influences (Cheverud et al. 1985; Cheverud and Routman 1995; Hallgrimsson et al. 2019; Morrissey 2015; Rice 2004; Wolf and Wade 2016). Mathematical models that predict variation based on the magnitude and timing of events along the life course are still fairly rudimentary, but they are producing predictions that are amenable to empirical challenges (Atchley and Hall 1991; Kavanagh et al. 2007; Machado et al. 2023; Rice 2004; Roseman and Delezene 2019; Vitek et al. 2020). These models may not be complex enough at present to provide satisfactory explanations at the level of granularity to satisfy everyone who wants highly detailed accounts of the relationships between development and evolution, but there is no doubt that these kinds of developments are taking place in the OS.

##### 2.2.1.2 Plasticity as Meta‐Adaptation

Stock et al. (2023) and other advocates for the EES in human evolution (e.g., Antón and Kuzawa 2017; Kissel and Fuentes 2021) argue that organisms have a propensity to display plastic responses to environments, even environments to which they and their ancestors have never been exposed, that are adaptive. In their account, humans have an enhanced capacity for this kind of adaptation. This goes beyond developmental plasticity and adaptive plasticity as they are defined in all branches of evolutionary theory, whether they be in the EES in human evolution, OS, or elsewhere. In this view, the enhanced disposition to produce adaptation through plasticity—the meta‐adaptive capacity—is itself an adaptation.

The argument that adaptive plasticity is increased in humans in a meta‐adaptive way is a radical extension of phenotypic plasticity as a mechanism for trait evolution. Plastic changes to human limb length that result in benefits to survivorship and reproduction in extreme climates that had not hitherto been encountered by the ancestors of individuals who moved into a novel environment, for example, would be a product of meta‐adaptation. In this case, developmental processes would allow individuals to attune their limb lengths to local conditions. That plasticity is a property of many aspects of organismal form is certain in that genotypically identical individuals regularly develop different phenotypic states under different environmental conditions. But it is an entirely different, and much more onerous undertaking to establish that plasticity tends to bias phenotypic outcomes such that they adapt organisms to environments—even those environments not previously experienced by a lineage—and is an evolved feature of some organisms and not others, as proposed by proponents of the EES in human evolution.

While we already argued that humans are unexceptional with respect to the magnitude of variation in body form, it is still possible that some of the variation in human body form is attributable to plasticity and perhaps adaptive plasticity. Were developmental plasticity the dominant mechanism behind human variation, we might expect that, in the case of human groups migrating from one location to another, their phenotypic states would quickly become disassociated with those of their ancestors. Sampled groups of humans do retain at least some morphologies closer to the mean of their ancestral groups and those groups to which they are related through common ancestry and gene flow (Auerbach 2010; Auerbach 2012; Katz et al. 2016; Roseman 2016; Roseman and Auerbach 2015; Temple et al. 2008). Plastic responses to different habitual behaviors associated with modes of subsistence appear to affect aspects of craniomandibular form, but this does not completely mask the tendency for group‐level differences to reflect gene flow and neutral divergence (Betti et al. 2009; Katz et al. 2017). While plasticity is apparent, it does not dominate over other sources of variation within and among groups.

That plasticity occurs, however, does not necessarily mean that the resulting phenotypic states are adaptive (see, e.g., arguments about how pelvic plasticity does not relate to selection in Auerbach et al. 2018). Humans are remarkable among mammals in that they live across the large majority of the land surface of the earth. Other than wolves, no terrestrial mammal comes close to having such an expansive range. A claim made in several EES in human evolution papers is that the plasticity of body form in humans has been key to being able to live in such a wide range of environments. Stock et al. (2023), for example, interpreted the work of Serrat and various colleagues (Serrat 2013; Serrat et al. 2008, 2014) on mice as evidence in favor of developmental plasticity being a source of variation in limb segment length in humans (few other studies have examined this experimentally; cf., DeRousseau and Reichs 1987). Serrat et al. (2008), Serrat (2013), Serrat et al. (2014) indicated that decreases in the amount of peripheral blood flow could have contributed to reductions in appendage length in mice raised in cold chambers (Serrat et al. 2008, 2014). Stock et al. (2023) suggest a similar mechanism adapts body form to climatic conditions in humans.

If temperature‐related plasticity is possible in mice and humans, the capacity for developmental plasticity might be a generic feature of mammals. While there is abundant evidence for broad patterns of ecogeographic variation in body form among closely related species (e.g., hares, passerine birds, bears), researchers of these taxa have not appealed to plasticity to explain differences in extremity length or body size among groups (Gigliotti et al. 2020; Luna‐Aranguré et al. 2020; McQueen et al. 2022; Nudds and Oswald 2007; Symonds and Tattersall 2010). Temperature has an effect on enzyme activity, binding affinities, and other biochemical properties, and absent of energetically expensive compensatory measures, cold temperatures might reasonably be expected to slow growth on thermodynamic grounds alone. Evolution might not enter into it. Rather than being an adaptive response, abbreviated limb length would be an indicator of a failure to thrive in a cool environment. We might as well call surviving for a long period of time after having lost a limb to a predator “plasticity,” which would rob the term of any explanatory power.

Also relevant to the issue of whether the plasticity we observe is adaptive, available evidence indicates that mammal populations migrate in response to increasing average annual temperature rather than adapting through novel variation produced through developmental plasticity (Buckley and Kingsolver 2012; Davidson et al. 2011; Davies et al. 2009; cf. Hetem et al. 2014). Even if temperature‐related phenotypic plasticity like that induced in the lab results in adaptive phenotypes, which is only just being investigated, it may not be sufficient to expand the range of habitable environments for organisms in the wild. One way or another, if there is an effect, we have neither the physiological nor biomechanical analyses to judge whether it would result in higher fitness. Serrat's (2013) (Serrat et al. 2012, 2022) experiments did not examine whether the plasticity observed displayed the kind of genotype by environmental interactive variation that would be required to allow for the evolution of adaptive plasticity. Without good models of the relationship between form and fitness combined with a robust understanding of the variational properties underlying the evolution of this kind, it remains unknown whether the plasticity reported in these experimental mouse models is informative about mechanisms by which other mammal appendages might vary across environmental gradients and whether any of it is adaptive.

This brings us back to meta‐adaptation. Adapting a population to an entirely unprecedented set of conditions, whether imposed on a group of organisms or co‐constructed as a part of its evolution, is a different matter that does not have much in the way of adaptationist theory to back it up. Outside of cultural evolution and the still speculative work on the evolution of the capacity for imagination, creativity, and foresight (Mithen 2001; Suddendorf and Corballis 2007; Zwir et al. 2022), the only theoretical work that might be able to explain this kind of generalized plastic response is distinctly non‐adaptationist in character. Flexibility inherent to modular gene networks seems to arise as a passive product of random evolutionary processes in complex organisms, even without much in the way of adaptation by natural selection (Lynch 2007; Lynch and Hagner 2015). Likewise, the evolution of novel adaptive characteristics that build on existing features can have a latent capacity to revert to the previous functionality, leading to a degree of phenotypic plasticity in a non‐adaptive fashion (Grant et al. 2021; Härer et al. 2017). All of this involves a relaxation of the efficacy of natural selection in small groups so that small differences in fitness imparted by slight variations in biomechanical performance or biochemical efficiency cannot overcome the effects of random genetic drift (Lynch 2012, 2020). Thus, the general tendency for complex multicellular organisms to exhibit plastic responses to their environment, even in superficially adaptive ways, may be a product of a *lack of adaptation* as opposed to a meta‐adaptation.

##### 2.2.1.3 Historical Contingency and Path‐Dependence

Proponents of the EES in human evolution (e.g., Prentiss 2021; Stock et al. 2023) make a point of claiming that systems of cultural inheritance playing out over evolutionary time scales lead to results that are structured in historically contingent ways. For example, Stock et al. (2023, 216) note, “culture [as an inheritance system] is historical and path‐dependent and can thereby create different adaptive or maladaptive trajectories for different groups, even when they live in comparable environments.” None of these studies in the EES in human evolution, however, deploy explicit theory or method to study historical contingency and path‐dependence in evolution, though these methods are well‐established within the framework of the OS.

To illustrate what we mean by historical contingency, a problem we investigated in some depth previously (Roseman and Auerbach 2015), we use the analysis from Stock et al. (2023) because it is one of the few applications of the EES in human evolution ideas to data. But readers should understand that the comparative approach they use—the strategy of grouping humans for the purposes of exploring evolutionary processes using linear statistical models—is a common practice in biological anthropology. In their fig. 4, Stock et al. (2023) present a depiction of the medians and box and whisker plots for estimates of the crural index for five age groups of subadults (intervals of 0–2, 2–4, 4–7, 7–12, and 12–17 years in age) in each of three geographically defined groups of foraging peoples sampled from the archeological record. The first geographically defined group is a composite of individuals from multiple locales within ±50° of the equator. The remainder are drawn from either representatives of the Kitoi culture in Siberia (at 53° N) or the Sadlermiut/Sallirmuit culture in Hudson Bay (at 64° N). The group compositions are displayed in their Table 1. By visual inspection, crural indices in the youngest subadults appear to be higher than in older subadults in the Kitoi and Sadlermiut samples while they seem uniform in the sample pooled from within ±50°. This is taken by the authors as an indication of plasticity in limb segment proportions arising from being exposed to extremely cold environments.

The results depicted in Stock et al.'s fig. 4 could be subject to multiple historical contingencies and path dependencies mediated by cultural and biological processes alike. We focus on two possibilities—ontogenetic change and population history/structure. Stock et al. (2023) correctly note that understanding ontogenetic change is an important component of understanding the causes of morphological change over evolutionary time. In this spirit, they cite the apparent decrease in the crural index with age in high‐latitude groups. Since each individual was measured only in postmortem skeletonized form and not at points along their development during life, their ontogenetic argument supporting a plastic response to an extreme environment treats cross‐sectional data as though it were longitudinal. That is, they interpret variation across differently aged individuals as though they were tracking change within individuals across ages.

It may be that the difference in the age structure of the crural index between the middle and extreme latitude groups is attributable to plastic changes induced by exposure to different prevailing temperatures. A simpler explanation, however, is that we are looking at a record of selection through viability that is either mediated by crural index or something with which it is correlated. That is, the limb proportion differences between the age classes might be attributable to a tendency for individuals with lower crural index to have higher early‐age survivorship than those with higher crural indices. Some combination of these factors and many other possibilities beyond them might also explain the pattern. In such a data‐depauperate situation in which much of the relevant context is impossible to apprehend, we are unlikely to come to any satisfactory conclusions.

Historical contingencies and path dependencies on evolutionary time scales are reflected in the spatial and temporal variation among groups produced by natural historical events and the evolutionary dynamics they induce (Gonzalez‐Voyer and von Hardenberg 2014; Stone et al. 2011). In this context, the evolutionary dynamics that are important are the ways in which gene flow, random genetic drift, mutation, and recombination produce a neutral variational background on which evolution by natural selection takes place.^2^ Understanding this neutral variational background is important both because it is a part of evolution and because it sets up conditions under which a single‐minded focus on adaptation can lead to profound misinterpretation of evolutionary outcomes (Butler and King 2004; Felsenstein 1985; Housworth et al. 2004; Roseman and Auerbach 2015). Sampled groups with variation disposed by gene flow and neutral evolution structured by shared common ancestry do not represent independent draws from a distribution of evolutionary outcomes (Katz et al. 2016; Roseman 2016; Savell et al. 2022; Stone et al. 2011). This problem is widely appreciated in the phylogenetic comparative literature, in which researchers continue to develop methods to address the non‐independence of lineages when modeling evolution (Felsenstein 1985; Nunn 2011; Uyeda et al. 2018).

Viewed with these hazards in mind, the comparisons featured in fig. 4 of Stock et al. (2023) to make the case for a plastic response to extreme cold are interpreted under the assumption that all groupings are independently evolving from a single common ancestor and thus treat them as if they were evolutionarily independent. Moreover, all individuals sampled within ±50° latitude are assumed to make up a single panmictic group in this comparison. That is, it assumes individuals from as far apart as central Africa and Australia were drawn from a single randomly mating group. As such, this EES in human evolution framing of the issue assumes a patently false evolutionary history over the relevant span of time.

The consideration of population history is essential in any evolutionary study. Traditional human comparative biology, including the ecomorphological investigation of skeletal form, has typically used ordinary least squares regression to test for associations between environmental and spatial variables on one hand, and aspects of skeletal form on the other (e.g., Holliday 1997; Pearce and Dunbar 2012; Ruff 1991). This application of a generic statistic to an evolutionary problem introduces the assumption, as we noted above, that groups have evolved independently of one another since all groups shared a common ancestor at the same time (Felsenstein 1985). Local groups of humans have not been evolving independently because of gene flow and their complex networks of common ancestry. The results of evolutionary theory informed investigations into human morphological variation confirm that neutral evolutionary processes structured by random genetic drift, gene flow, and neutral mutation make strong contributions to the distribution of human form (Betti et al. 2012, 2013; Hallgrimsson et al. 2019; Katz et al. 2016; Mallard and Auerbach 2020; Roseman 2016; Roseman and Auerbach 2015; Savell et al. 2022; Schroeder and von Cramon‐Taubadel 2017; von Cramon‐Taubadel 2019). The consequences of natural selection and group‐specific environmental influences are apparent on the among‐group differences, but selection is clearly not nearly the all‐powerful driver of the evolution of human diversity today and in the recent past that the methods of ecomorphology and human comparative biology implicitly assume. The lesson learned from studies of human variation like these is that we cannot model how evolutionary processes shaped trait evolution based on comparisons of trait means that do not incorporate the historical contingency and path dependencies that are inherent to evolution, be it biological or cultural.

#### 2.2.2 The Evolution of Large Brains in the Genus *Homo*

We now focus on the second case study for comparing how human evolution is examined using ideas from the EES with approaches derived from the diverse literature that falls under the OS. As in the example above, examining human body form, the second case also focuses on plasticity as an alternative to selection. The evolution of large brains in humans over the last 2 Ma has attracted considerable scholarly attention throughout the history of the study of human evolution (e.g., Du et al. 2018; Gingerich 2022; Grabowski 2016; Navarrete et al. 2011; Pilbeam and Gould 1974; Rightmire 2004; Tobias 1987). Historically, accounts of brain size tend to be post hoc adaptationist accounts that try to relate the increase in brain size, usually measured as endocranial volume (ECV), to the evolution of certain cultural practices thought to necessitate extra cognitive capacity that required additional brain expansion (Stanley 1992; Tattersall 2018; cf. Ponce de León et al. 2021). Other explanations include adaptation to physical environments such as the heat of the Pleistocene African savannah (Falk 1990).

Antón and Kuzawa (2017) present an explanation of the evolution of large brain size in the genus *Homo* that features adaptive plasticity as the important driver. Their flexible‐stem model (FSM) proposes that when an ancestral (stem) population is faced with new ecological pressures, the pattern of phenotypic plasticity in the ancestor will constrain the direction and form of phenotypes induced in descendant populations. Taking the claim that there is a high degree of variation in brain size in *H. erectus* as their empirical point of purchase on the problem, they argue that this purported increased variation “point[s] to an unusually high level of variability in cranial capacity, which could either point to a predominant role of genetic selection on these traits, or perhaps more speculatively, to relatively greater plasticity in brain size in this species than what is observed in contemporary humans” (Antón and Kuzawa 2017, 10). According to this reasoning, greater variation on a unitless scale, as represented by the coefficient of variation (CV) could be an indicator of the degree to which environmental effects related to the conditions under which an organism develops may influence development.

This framing of the issue of brain size evolution allows us to articulate two questions that can serve as a test on the OS and EES in human evolution theory and allows us to directly compare their explanatory capacities. In the first, we ask whether the rate of brain size evolution was too fast to be explicable using evolutionary genetic models from the OS. If so, it might provide a warrant for drawing on the EES in human evolution tools to investigate a situation that is extraordinary and perhaps out of the domain of applicability of OS theory and technique. Second, we test whether human brain size variation is exceptional among primates and mammals as this is a warrant for calling for an EES in human evolution‐based approach under the supposition that more standard theories cannot accommodate such high levels of variation.

##### 2.2.2.1 Was Human Brain Size Evolution Too Fast to Be Accounted for by OS Theory?

The focus on magnitude of ${\mathrm{CV}}_{\mathrm{ECV}}$ values in the empirical applications of the FSM implies that additional plasticity‐induced variation is necessary for *ECV* evolution in *Homo* because of some rate‐limiting effect of variation on evolution. That is, if the evolution of brain size is remarkably slow relative to other morphological traits, then plasticity might have provided a boost. We can place bounds around our expectations for rates of morphological evolution using the neutral theory of phenotypic evolution and comparisons of phenomenological models to express expectations for different modes and tempos of evolution. At first glance, rates of evolution of brain size in the genus *Homo* through the Pleistocene seem quite rapid, in line with the framing of the problem presented in Antón and Kuzawa (2017). However appealing and widespread the intuition might be that the rates of *ECV* evolution are high, the EES in human evolution lacks a theoretical framework that might structure questions about the rates and causes of evolution, and does not provide an appropriate analysis of variation. But theory and methods stemming from quantitative genetics in the OS provide a means of analysis.

Gingerich (2022) analyzed a near‐comprehensive dataset of *ECV* estimates for humans spanning the last 2 Ma and concluded that there were two periods of strong directional trends of increasing brain size separated by a brief period of non‐directional evolution caused by random genetic drift or a lack of evolution (as one might expect under stabilizing selection). The per‐generation rate of directional evolution was very high, about 0.15σ per generation on the phenotypic scale (i.e., 0.15 phenotypic standard deviations), and comparable to rates of evolution observed in short‐term responses to natural selection in natural contexts (Gingerich 2019).

A theoretical examination of evolutionary rates buttresses the empirical observation that the rate of *ECV* evolution is high. Theory developed in the OS—the neutral theory of phenotypic evolution—provides a way of constructing a distribution of evolutionary outcomes in the absence of the action of natural selection given estimates of the average amount of genetic variation in the population through time (given here by evolvability, symbolized as *e*), effective population size (*N*_*e*_), and the amount of time (*t*) in generations. In our case we use *e* = 0.009 (Miller and Penke 2007), *N*_*e*_ = 5000, and $t=7.4\times 10^{4}$, which puts the human generation time at 29 years (Langergraber et al. 2012). On the evolvability and natural logarithmic scales, we can express Lande's (1976) treatment of the variance of the among‐population distribution of neutral evolutionary outcomes as $\sigma_{B_{\mu }}^{2}=\frac{\mathrm{et}}{N_{e}}$. This expression reflects the fact that, for a given *t*, evolutionary outcomes under random genetic drift have higher variance when the population size is small (stronger drift) or when there is more genetic variation in the trait (higher evolvability). Using estimates from Gingerich (2022), the long‐term evolution observed in human *ECV* registers a 3.4σ deviation from the neutral expectation using a constant‐heritability model ($p\left(x\geq 7.25\right)=4.6\times 10^{-4}$). Thus, hominin brain size evolution is well outside the bounds of the expectation under neutrality.

We can also build a neutral expectation using a mutation‐drift equilibrium model (Lynch 1990). The mean square standardized mutational variance of mass and body composition traits in mice is $\approx 4\times 10^{-6}$ (Houle et al. 1996). This treatment registers a 2.1σ deviation from the neutral expectation, which would be regarded as a statistically significant departure from the neutral expectation at α=0.05 ($p\left(x\geq 7.25\right)=0.019$). The discrepancy between the two results is typical in that mutation‐drift equilibrium predicts higher rates of neutral evolution than do pure random genetic drift models. With respect to whether the rate of brain size evolution is too rapid to be accommodated by the evolutionary theoretical state of the art, this result indicates that, while on the margins of what would be expected by neutral evolution and thus fast, it is not at all out of the ordinary.

We can also use Gingerich's figures combined with estimates of the rates of brain size evolution over different time scales to estimate the strength of directional selection needed to sustain the evolution of increasing brain size and compare them to estimates of the strength of selection observed in contemporary living groups. Rearranging the univariate evolvability rendition of the Lande equation to estimate the mean standardized selection gradient ($\beta_{\mu }$) as $\beta_{\mu }=\Delta {\bar{z}}_{\mu }/e$ and drawing on the evolvability figure we used when testing neutrality, we obtain estimates of the strength of selection needed to explain the rate of evolution of brain size at the hundreds over intervals of 40,000 generations and single generation time scales of $\beta_{\mu }=0.01$ and $\beta_{\mu }=1.67$, respectively. That is, the strength of selection on *ECV*—or more accurately, some combination of things that are correlated with and mediated by *ECV*—is 0.01 to 1.67 times as strong as it was on fitness itself depending on the time scale.

Estimates of the strength of selection on body mass and morphological traits from studies of wild populations are typically much lower on the generation to generation time scale than the value we obtain, averaging around 0.3 once adjusted for elasticities of fitness (following Hereford et al. 2004, “elasticity” describes the proportional degree to which a quantity like fitness will change given a standardized change in another quantity like brain size). While strong in comparison to the average, our single generation time scale estimate is comfortably accommodated in the observed distribution of outcomes. The strong selection for larger brain size arrived at using an estimate of the rate of evolution on the generational time scale was not sustained over hundreds of thousands of generations. Rather, the high generation to generation rate and the robust corresponding strength of selection includes a good deal of jittering back and forth as the rates were cast in absolute terms. The $\beta_{\mu }=0.01$ per generation strength of selection arrived by estimating rates over 40,000 generation time intervals is slight by the standards of those estimated in groups of wild organisms and would likely not be detected using conventional methods (Hereford et al. 2004). It is important to point out that reckoning rates on an absolute scale imparts an upward bias to rate estimates that carry over to the estimates of $\beta_{\mu }$ (Hereford et al. 2004). Thus, our estimates of $\beta_{\mu }$ are likely inflated which further substantiates our claim that the range of magnitudes of selection needed to evolve large brains during the Pleistocene is *not* out of the ordinary.

The present results and those of similar analyses rooted in the OS (e.g., Schroeder and von Cramon‐Taubadel 2017) allow us to rule out wide swaths of the space of evolutionary possibilities. Neutral evolutionary change and strong stabilizing selection around a constant optimum in the absence of directional selection are unlikely to have produced the observed changes. Our results accord well with the conclusion of Du et al. (2018), who noted that the evolution of large brain size was probably more a matter of human groups tracing a shifting optimum through time rather than directional selection toward a very distant static optimum. Likewise, the evolution of brain size is not a product of a correlated response to selection acting through body size, nor is the evolution of the two traits independent processes. Rather, it is more likely that the evolution of increased body size was driven by a correlated response to selection acting on brain size (Grabowski 2016). Further refinements to the OS‐based analysis of the rates of brain size evolution and the dynamics of evolutionary change might include phylogenetically informed analyses to obtain lineage‐specific estimates of the relevant parameters and take into consideration uncertainty in dating, phylogenetic structure, and the effects of hybridization.

While the results we report and review give us good evidence that natural selection was involved in the evolution of large brains, it does not give us so much as a wisp of a clue as to what the conditions of life were like to cause the covariation between differential reproductive success and brain size through time that would allow natural selection to occur. Our intuition that it probably was not brain size itself is just that, intuition, and does not even register as an untested hypothesis because there is no method by which we could test it. Understanding the relationships between behavior, brain form, function, and fitness that would be required to substantiate a claim of adaptation is a much more difficult issue. With respect to our question about whether the rates of *ECV* evolution in humans through the Pleistocene are rapid enough to warrant the construction of new basic evolutionary theory, the answer is a clear “No.”

##### 2.2.2.2 Is Variation and Variability in Brain Size During Human Evolution Exceptional?

While strong, the strength of natural selection needed to evolve large human brains through the Pleistocene is not implausibly formidable. The conclusions drawn in the previous analysis, however, do not allow us to say whether the *variational* properties of brain size during human evolution were unusual. The FSM predicts that *Homo erectus* should be more phenotypically plastic than other members of the genus *Homo* and other primates alike, and this increased plasticity will be evident in comparatively higher coefficients of variation values for endocranial volume. While Antón and Kuzawa present estimates of *CV*_*ECV*_ from groups in the genus *Homo* spanning various amounts of time ranging from the hundreds of thousands of years time scale to a catastrophic assemblage deposited in a geological instant, they do not provide either a statistical comparison of the magnitudes of *CV*_*ECV*_ or a comparison to a distribution of *CV*_*ECV*_ estimates drawn from a range of other species to evaluate their claims.

A comparison of a set of fossils collected from across multiple continents spanning a temporal range of hundreds of thousands of years with a skeletal sample gathered from mortuary contexts in the recent past is not going to be informative. The fossil sample will display variation that represents evolved differences in space and time that are not representative of the local variation at any one point in time that will form the grist for the evolutionary mill. We can then establish a sense of the range of uncertainty in the estimates of the differences between groups to help us decide if the difference we observe is a genuine biological phenomenon. The differences between the ${\mathrm{CV}}_{\mathrm{ECV}}$ estimates for *H. erectus* samples highlighted in the main investigation of the FSM (Antón and Kuzawa 2017) and the recent human group with the lowest level of variation have high uncertainty. Their 95% confidence intervals overlap zero (Dmanisi vs. Spitalfields ΔCV=3.8%, 95%CI: −2.2% to 9.8%; Ngandong vs. Spitalfields ΔCV=1.0%, 95%CI: −3.9% to 5.9%).

**FIGURE 1:**
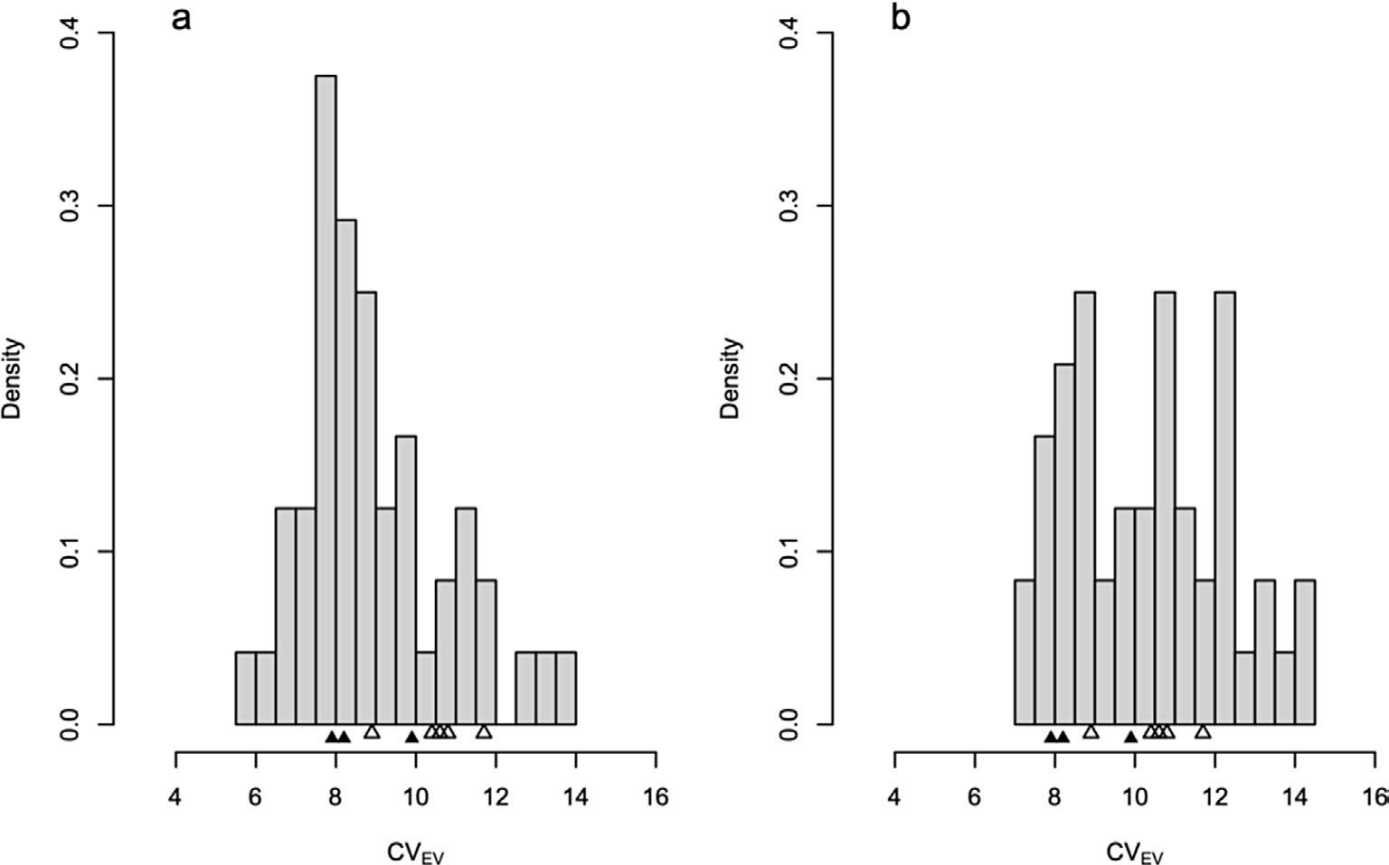
Drawing on the primate order‐wide data presented by Isler et al. (2008), we estimated the values for pooled within‐sex variation to control for sex differences and for total variation that includes variation from sex differences for 48 species (Table). Plotting the sex‐corrected and full‐sample *CV*_*ECV*_ using histograms (panels a and b, respectively) and indicating the values for the non‐sex‐corrected *CV*_*ECV*_ for *H. erectus* and *H. sapiens* from Antón and Kuzawa (2017) using marks below the distribution demonstrates that neither *H. erectus* nor *H. sapiens* is at all unusual when it comes to *ECV* variation among primates. This is particularly apparent in the non‐sex corrected treatment (panel b), which is the most apt comparison given that the Antón and Kuzawa figures include variation arising from sex differences.

A broader comparative perspective demonstrates that *ECV* variation in genus *Homo* is wholly unremarkable. Estimates of *ECV* from across a range of primate species (data from Isler et al. 2008) show that neither *H. erectus* nor *H. sapiens* is at all unusual when it comes to *ECV* variation among primates (Figure 1). Since *ECV* in *H. erectus* does not vary appreciably more than in other members of the genus *Homo* and genus *Homo* displays wholly unremarkable *ECV* variation when compared to other primates, there is no empirical warrant for an appeal to the special influence of plasticity in an evolutionary explanation of the evolution of large *ECV* in humans, or to appeal to plasticity to explain evolution across primate species.

Human *CV*_*ECV*_ also tends to be smaller than *CV* estimates for similar volumetric and mass traits in other organs and tissues in the human body (Miller and Penke 2007). This might indicate that *ECV* is more canalized against developmental perturbations of any kind (genetic, environmental, or interactive) than other volumetric traits in humans. Without experimental models that demonstrate the mechanisms behind these potential discrepancies, we can only speculate about causation. One hypothesis may be that the smaller *CV*_*ECV*_ relates to the documented phenomenon of intrauterine brain sparing, where the tendency is for brain growth to be maintained at the expense of other tissues when a developing organism is energetically constrained (Baker et al. 2010; Barbeito‐Andrés et al. 2019). The observational and experimental designs used to study purported brain sparing do not, thus far, provide dispositive results.

Theoretically speaking, it is also not clear that the second moment—the variance and other quantities based on variance such as the standard deviation, evolvability, or coefficient of variation—is the property of the distribution that should be evaluated. Plasticity‐driven changes would be evident in the generation‐to‐generation shifts in means and not necessarily in the variance (i.e., the second moment of the distribution of phenotypic values). Moreover, higher variation in and of itself will not increase the rate of evolution. To facilitate the response to directional selection, the increase in variation would have to be through an increase in the genetic, or otherwise appropriately heritable, variation. But this is not part of the written descriptions of the plasticity‐driven models of evolution presented by Antón and Kuzawa (2017) and Stock et al. (2023). If there was a tendency for plasticity to produce variation in an adaptive direction, that is, in the direction of larger brains or relatively shorter tibiae, it would present itself as skewness of the distribution. No study we have read in the EES in human evolution considers this. This is probably just as well, as the power to detect changes in skewness is lower than the power to detect changes in variance. As such, it is unlikely that this issue will be resolvable until substantially more fossils are discovered, especially fossils from assemblages of individuals unambiguously from the same narrow time period. Alternatively, one might see a plastic shift in the mean for a characteristic, which is not unusual for complex traits. Maintaining a secular trend for millions of years in what appears to be a highly canalized trait, however, would depend on developmental and trans‐generational processes that are left to the reader's imagination in the EES in human evolution literature.

Whether in the case of heightened variation or in cases of skewness or mean shifts in putatively adaptive directions independent of evolution by natural selection, these scenarios would involve producing novel plastic changes that have not previously occurred in that lineage. The evolution of large brains well beyond the bounds of what is observed in ancestral groups poses a problem for the evolution of adaptive plasticity because the well‐worked out theories—all of which have their origins in the OS—show that the evolution of adaptive plasticity tends to take place when there is both sufficient genetic variation in plasticity itself and in an environment that varies in time or over the spatial range in which organisms might find themselves in a lifetime (Lande 2009, 2014; Sommer 2020). That is, we expect adaptive plasticity to evolve within a range of environments to which a lineage has been repeatedly exposed over a sufficiently long time. What is clear enough, however, is that the invocation of massive adaptive plasticity recommended by the EES in human evolution is wholly unfounded even when evaluated on the standards of its own prescription.

### 2.3 The OS Gives Us the Tools to Study Evolution, Whether or Not That Involves Plasticity and Adaptation

While the OS has theory to deal with plasticity and extended systems of inheritance as opposed to the EES in human evolution, which is devoid of such conceptual tools, the OS also stands apart from the EES in human evolution in that it is capable of articulating questions about a wide range of evolutionary dynamics in historically contingent and path‐dependent scenarios. That is, the OS equips us to take natural selection and adaptation to be a part of our questions as opposed to being the only framework for constructing an explanation. In our review, we have found that studies in the EES in human evolution, on the other hand, are focused within an adaptationist framework. Though EES in human evolution studies make passing mentions about the importance of neutral processes in evaluating the evolution of traits (e.g., Stock et al. 2023, 212), these studies do not incorporate it into the framework by which they evaluate evolution.

As we demonstrated in our reevaluation of the evolution of limb proportions and brain size, theory in the OS offers models with organic connections to methods that we may deploy to connect theory with data to yield informative results. By “informative” we mean results that provide accurate estimates of relevant parameters with a clear sense of their precision; are capable of distinguishing between different models and thus able to rule out sections of the vast possibility space of evolution; and can make use of causal inference to draw conclusions about the workings of evolutionary dynamics. The results of a study statistically characterizing the relationship between aspects of morphology and climatic variables that are putatively involved in natural selection while simultaneously accounting for historical contingencies is a case in point (Cho et al. 2022; Felsenstein 1985; Housworth et al. 2004; Katz et al. 2016, 2017; Roseman and Auerbach 2015; Savell et al. 2022; Stone et al. 2011). There are relationships between climate and morphology that are consistent with the action of natural selection or plastic response to local conditions. *However*, they are not nearly as strong as previously thought and random genetic drift and gene flow have a strong effect on worldwide variation in skeletal form (e.g., Savell et al. 2022). Likewise, it is apparent that much of human morphological evolution over the Pleistocene and Holocene has been a product of random genetic drift and not a set of responses to natural selection (e.g., as suggested by results in Grabowski and Roseman 2015 and in Roseman and Auerbach 2015). Comparisons of models that include a term for the plastic response of facial and mandibular bone to mode of subsistence fit better than models of evolution by random genetic drift alone, indicating that plasticity *is* a strong candidate for explaining some among‐group variation in skeletal form (Katz et al. 2017). That is, studies rooted in OS theory are the ones that are discovering actual plasticity. The EES in human evolution provides only speculation about plasticity.

In these OS‐based studies, researchers typically did not assume the operation of an evolutionary or other biological process at the outset (except, e.g., Grabowski 2016; Grabowski and Roseman 2015; Rolian et al. 2010; Savell et al. 2016, who were collectively interested in correlated and direct responses to directional selection as hypothesized by others). Rather, studies that used models to craft expectations and specific hypotheses (e.g., how neutral processes would shape variation) examined which model presented a better fit to the data than competing expectations or which hypotheses could be rejected using methods that explicitly include appropriate evolutionary theoretical structure. This sets the OS‐informed study of human evolution apart from other evolutionisms within biological anthropology, as the evolutionary theory in the majority of studies in human evolution (whether promoting the EES or other approaches) is wholly disconnected from statistical methods that permit the rigorous analysis of evolution. What passes for evolutionary theory in human evolutionary studies too often consists of a vocabulary and a set of qualitative notions that make their first appearance in the introduction, recede into the background for the methods and results, and then reappear to frame the discussion in a manner that has no formal relationship to the tests conducted. We, among others in recent years, have highlighted this concern (see, e.g., Auerbach et al. 2023; Rolian 2014; Schroeder and Ackermann 2023; von Cramon‐Taubadel 2019).

Lastly, OS‐based theory and method permit us to make tentative causal interpretations in evolutionary and developmental contexts. The ability to estimate the multivariate vector of directional selection demonstrates that the direction of selection might be quite different from the response to selection, in part because many aspects of organisms are genetically covariant and so cannot respond to evolutionary processes independent of each other (Agosto and Auerbach 2021, 2022; Grabowski 2016; Grabowski and Roseman 2015; Savell et al. 2016; Schroeder and von Cramon‐Taubadel 2017). That is, the traits that catch our attention by evolving quickly or in directions that pique our interest might not have been subject to directional selection. Distressingly for non‐process‐oriented ways of describing evolution such as we observe in the EES in human evolution, these characteristics might have been subject to selection acting in a direction *opposite* to the realized response (Hereford et al. 2004; Grabowski and Roseman 2015; Savell et al. 2016). Failure to account for the difference between natural selection and evolution by natural selection makes linking non‐neutral evolutionary processes to properties of organisms impossible.

The benefits of an OS‐based approach over its EES in human evolution counterparts include: (1) presentation of a unified and internally consistent treatment of variation, plasticity, and genetics and their relation to evolution; (2) breaking down of the dependence on adaptationist perspectives; and (3) the availability of well‐assessed tools to deploy to answer questions about evolution in complex non‐equilibrium contexts that feature historically contingent and path‐dependent events and influences. In both of the examples drawn from the human evolution literature, the OS provides clearer, more pluralistic, and more informative theory and methods than the EES in human evolution. It is apparent from the results of the competing analyses that we present here that the EES in human evolution cannot help researchers identify or generate good predictions, build appropriate tests for predictions, structure comparisons among models, or build models consistent with what is known about evolutionary processes (e.g., plasticity or heritability).

## 3 The Hazards of Anti‐Theory

To build a scientific program, we would need an internally consistent theory capable of making clear predictions and establishing clear connections to data through theoretically informed methods. We have demonstrated that the EES in human evolution has none of these necessary features. Moreover, it rejects scientific theory building. It does so by offering impressionistic and particularistic renderings of supposed complexity. Its insufficiencies actively preclude the construction of generalizable conceptual tools needed to prove that things are indeed complex and allow us to build explanations of substantiated complexity. As such, the EES in human evolution is a kind of *anti‐theory* that opposes abstraction and inference to the best explanation through analysis of data in favor of providing verbal impressionistic explanations.

We get a sense that the EES in human evolution literature is appealing because it superficially satisfies a concern that has been drilled into the collective wisdom of evolutionary science—that we should not be uncritical adaptationists (Gould and Lewontin 1979)—but does not require the additional training in evolutionary theory and methods required to incorporate all evolutionary processes and their technical details into formal models capable of producing explanations. The fact that the EES in human evolution does not require training in theory and technique that are often challenging and hard to master makes it easier to learn. Given the perverse incentives to grow enrollments and to publish quickly in academic settings, the ease of learning the rhetorical idiom of the EES in human evolution might very well crowd out more productive evolutionary approaches that require more extensive training to learn. All programs invested in training evolutionary theory should resist this trend and demand rigorous education in methods that we show to be better equipped to address evolutionary questions.

Beyond causing mischief within the literature of biological anthropology and other allied disciplines involved in the study of human evolution, the widening use of the anti‐theoretical EES in human evolution is ruinous to our ability to counter hereditarianism and race science, among other ideologically motivated scientific programs (Roseman 2018). While EES in human evolution proponents are vocal critics of hereditarianism and race science (e.g., Wells and Stock 2011; Fuentes 2021), these objections amount to mere rhetorical cover for what is a worldview, when critically examined, that is in full agreement with basic theoretical and analytical tenets of hereditarianism and race science (Roseman 2018). That is, proponents of the EES in human evolution regularly take “genetic” to mean “determined,” which is the foundational principle of hereditarianism and race science. They also adopt analytical frameworks that assume simple and false histories and are incapable of dealing with the known complexity of human evolution.

An example of the point we are making can be seen in the fact that studies used as evidence for plasticity and non‐genetic inheritance in the EES in human evolution (e.g., Kuzawa et al. 2010; Chung and Kuzawa 2014) have the same basic design as the family studies of hereditarians and race scientists in that both assume away the possible genetic causes of similarities among relatives—they are related, after all—in favor of interpretations that lean on structured environmental effects. Hereditarian and race science literature takes the same kind of study but assumes away the possibility that non‐randomly distributed environmental effects might account for the same similarities. Even the most cursory examination of the scientifically rigorous corners of the literature on familial resemblances in complex traits gives good reasons to doubt both positions (Roseman 2018). In either case, careful experimental or genetic epidemiological designs are required to answer questions about plasticity, genetics, or any other cause of familial resemblance.

A less obvious hazard of the increase in popularity of the EES in human evolution is its habit of simply stating that evolution and variation are complex without putting in the work of developing a theory of complexity or doing the groundwork to empirically demonstrate that something is complex by some set of meaningful criteria. The ritual incanting of “complexity” without regard for the theoretical, empirical, and methodological tools needed to define and measure complexity crowds out critical thought and gives cover for the acceptance or rejection of ideas based on esthetic and ideological standards independent of their explanatory value. This differs little from the kind of scientism that proponents of the EES in human evolution decry when it is deployed by hereditarians, race scientists, and evolutionary psychologists to translate their work into social policy. Combatting the pernicious influence of biological determinism and racial essentialism on public life requires better theory.

## 4 What Theory Should We Be Using and Where Can We Get It?

Where most discussions about the place of the EES in the larger evolutionary milieu tend to focus on whether it is novel or necessary, we instead evaluated whether the EES in human evolution can structure a productive research program for the study of human evolution. Using head‐to‐head comparisons of the performance of theory and method from the EES in human evolution and that from the wider OS, we demonstrate that theory and methods of the OS better equip us to formulate questions and design research programs to evaluate them by a large margin. Through a critical examination of the EES in human evolution, we demonstrate that it harbors notions of genetics, plasticity, heritability, and adaptation that are out of step with current biological science. On each subject, we articulated an affirmative account of what the OS can do for researchers in terms of guiding the building of our understanding of *evolution* irrespective of whether it involves adaptation in ways that the EES in human evolution cannot. To be perfectly clear, it is not that we reject the importance of plasticity or processes of evolution beyond selection. Rather, it is that we are convinced that the theory and the method we use from the OS are capable of structuring a productive research program on plasticity and processes of evolution beyond selection that those provided by the versions of the EES used in human evolution cannot.

Adopting EES‐based theory and methods that do not include, or even explicitly reject, the well‐developed and ever‐changing principles of the OS carries with it a pernicious hazard: by dispossessing us of tools to distinguish between evolution that is genuinely complex vs. a situation in which there is just a lot going on, we risk telling elaborate stories about noise. If a theory provides only another idiom in which to tell stories about adaptation and weave triumphalist narratives about how *Homo sapiens* became the sole remaining hominin species, it will not provide the kind of conceptual framework the field needs to study human evolution.

The good news is, as we have demonstrated here, advances in theory developed in the OS are already being applied in human evolution by many scholars to pose and answer evolutionary questions. Ranging from applications of neutral theory to test hypotheses about the dynamics of evolution, to the merging of developmental and genetic perspectives, on to how plastic responses acclimate humans to environmental change, the study of human evolution as a part of the OS has seen a healthy growth in the direction of advancing the study of questions the EES in human evolution says the field ignores. Add to this the advances made in the application of evolutionary theory to problems in culture, including its interaction with genetics, and we are presented with a vibrant and theoretically variegated research program rather than a stale and rigid orthodoxy.

The body of theory and method of the OS can be difficult for those outside of evolutionary biology or several of its closely related fields to obtain. A good way to get started is to attend workshops held by experts and to supplement your in‐person experience with freely available online resources. Table 1 contains a selection of the available workshops and resources that we recommend, with the proviso that these resources are often changing and growing.

**TABLE 1:** Workshops and online resources to help you learn more about evolutionary modeling and methods.

Workshop/online resource	Topics covered	Website
Evolutionary Quantitative Genetics Workshop	Evolutionary quantitative genetics and phylogenetic comparative methods	https://fhl.uw.edu/courses/course‐descriptions/course/evolutionary‐quantitative‐genetics‐workshop/ https://eqgw.github.io/
Sydney Phylogenetics Workshop	Phylogenetic comparative methods	https://meep.sydney.edu.au/workshops/
European Research Group that networks researchers in ecology, genetics, genomics, and evolution	Online courses and summer programs in various topics related to the four fields	https://www.evoltree.eu/
American Society of Human Genetics workshops	Various workshops on quantitative and population genetics organized on *ad hoc* basis (most are recorded)	https://www.ashg.org/
National Institute for Modeling Biological Systems (NIMBioS)	Multiple workshops and tutorials on evolution, biological modeling, and genetics are offered (most are recorded with materials provided)	https://www.nimbios.org/tutorials/
Graham Coop's population genetics notes from *Population and Quantitative Genetics*	Illustrations and examples drive this online publication of population and quantitative genetics	https://github.com/cooplab/popgen‐notes/

The OS offers students of human evolution an abundantly effective and ever‐growing framework with which to structure investigations. The unusual problems faced in human evolution provide ample opportunities for a more theoretically active study of human evolution to make novel contributions to evolutionary theory as part of the OS as a whole. Such interdisciplinary engagement will strengthen all evolutionary fields.

## Data Availability

All data used in this paper were drawn from the existing liter.

## Endnotes

^1^
“Body form” includes stature, body mass, and the relative lengths of distal and proximal limb elements such as crural indices (tibia/femur length).

^2^
This is not to say that evolution by natural selection acts independently of other evolutionary processes.
